# Mediolateral Margin of Stability highlights motor strategies for maintaining dynamic balance in older adults

**DOI:** 10.1371/journal.pone.0313034

**Published:** 2024-10-31

**Authors:** Tarique Siragy, Yuri Russo, Brian Horsak

**Affiliations:** 1 Center for Digital Health & Social Innovation, St. Poelten University of Applied Sciences, St. Pölten, Austria; 2 Public Health & Sport Sciences, University of Exeter, Exeter, United Kingdom; 3 Institute of Health Sciences, St. Poelten University of Applied Sciences, St. Pölten, Austria; Northumbria University, UNITED KINGDOM OF GREAT BRITAIN AND NORTHERN IRELAND

## Abstract

The dynamical nature of gait increases fall risk for older adults as the Center of Mass (COM) is constantly displaced inside and outside the Base of Support (BOS). Foot placement and leg joint moments are the primary mechanisms controlling dynamic balance. The Margin of Stability (MOS) quantifies the distance between the COM dynamical state and the BOS. While research examined how aging affects the relationship between foot placement and MOS, the relationship to leg moments is unexamined. Examining this relationship would elucidate whether aging increases fall risk from changes in the joint moments controlling the COM. Fourteen older (66.9 ± 4.3 years) and sixteen young (26.3 ± 3.6 years) adults walked along a 12m path for three trials. The MOS, hip and ankle moments in sagittal and frontal planes were analyzed. For the knee, only the sagittal plane was analyzed. MOS was calculated as the distance between the extrapolated-COM and the Center of Pressure per step. Statistical Parametric Mapping independent t-tests assessed group differences. Cross-correlation quantified MOS and joint moment relationships per plane during single-stance. No group differences in walking speed were observed. A larger frontal plane MOS, hip abduction and ankle eversion moment occurred for older adults. Cross-correlation demonstrated moderate and strong relationships for the hip-MOS for both groups in the sagittal plane. Older adults had a larger sagittal plane hip-MOS correlation than young adults. The larger mediolateral MOS in older adults may indicate attempts to avoid lateral balance loss by shifting their COM away from their BOS lateral boundaries during single-stance. However, this strategy moves the COM toward the BOS medial borders potentially pre-maturely terminating the contralateral swing phase during medial destabilization. The stronger sagittal plane hip-MOS relationship in older adults may reflect increased coupling between hip moments and the COM to control dynamic balance.

## Introduction

Approximately one-third of all older adults fall annually with most falls occurring while walking [1–4]. After the initial fall, the risk of sustaining a subsequent fall within the following six months rises drastically which poses a significant medical and socioeconomic threat for the victim while placing substantial monetary strains on healthcare systems [2–4].

The risk of falling while walking is relatively high for older adults due to the dynamical nature of this motor task [5–7]. During the single-support phases, which together account for 80% of the gait cycle, the Center of Mass (COM) is displaced and held in front of the foot which defines the surface area in which the Center of Pressure (COP) can act [6, 8, 9]. This in turn defines the Base of Support (BOS) [6, 8, 9]. Additionally, during the double-support phases, despite the COM being held inside the BOS, stability is challenged as the velocity of the COM is redirected laterally from the unloading to the loading leg [6–9]. Thus, the ability to maintain stable motion of the COM while it is in a constant dynamical state during walking is termed dynamic stability [5–9].

Research suggests that the two primary neuromuscular mechanisms that control dynamic stability are foot placement during the double-support phase and joint moments generated by the stance leg during single-support [6–10]. To avoid a fall at upcoming initial-contact, the neuromuscular system integrates sensorimotor information about the position and velocity of the COM during the stance phase to determine effective foot placement of the contralateral swinging limb [6, 11–13]. After foot placement is established, the neuromuscular system then corrects for any potential errors by adjusting COP underneath the foot [6, 9, 10]. In contrast, during single support, dynamic stability is maintained by the ankle, knee, and hip joint moments in the sagittal plane as well as by the hip and ankle moments in the frontal plane [6, 10]. Specifically, the leg in the single-support phase generates these moments to counterbalance the destabilizing forces acting on the COM from the hip joint reaction force (sagittal plane) and the force of gravity (frontal plane) [6, 9, 10].

Healthy aging, however, affects both neuromuscular mechanisms. Specifically, older adults walk with wider, shorter, slower, and sometimes more variable (in the case of older adults who fall) steps, have a stronger coupling between COM motion and step width, and walk more asymmetrically than young adults [5, 11, 12]. Additionally, a distal-to-proximal redistribution of leg joint moments occurs whereby older adults, compared to younger, walk with relatively greater contribution from the hip than ankle moments [14]. This redistribution of joint moments may impact the control of dynamic stability during the single-support phase as the relative contribution of the hip moment in controlling the COM may increase in healthy older adults. While research has quantified how healthy aging affects the relationship between foot placement and dynamic stability, a quantification on how aging affects the relationship between leg joint moments and dynamic stability remains unexamined [5, 12]. As the single-stance phase accounts for most of the gait cycle and has the narrowest BOS, examining the impact of how healthy aging affects the relationship between leg joint moments and dynamic stability would elucidate whether older adults are at an increased fall risk due to changes in the forces controlling the COM.

To quantify dynamic stability, and potential aging effects thereon, research commonly utilizes the Margin of Stability (MOS) [5, 15]. The MOS quantifies the distance of the extrapolated COM (combined term of COM position and velocity) to the edges of the BOS in the sagittal and frontal planes at discrete time points in the gait cycle [5, 15]. While current evidence demonstrates that older adults walk with a reduced anteroposterior MOS (MOS-AP), relatively few studies have examined how aging affects the mediolateral MOS (MOS-ML) during unperturbed overground walking despite older adults having increased instability in the frontal plane than young adults [5]. Moreover, the majority of research thus far has predominately quantified the MOS only during initial-contact or toe-off which only reflects how adjustments to foot placement affect dynamic stability [5, 16–22]. Critical information on healthy aging may be lost when quantifying the MOS only at specific points instead of continuously over an entire step. Additionally, by quantifying the MOS continuously, how aging affects the relationship between leg joint moments and dynamic stability during the single-support phase can be examined.

Thus, the purpose of this article is to examine whether healthy aging affects the relationship between the MOS and leg joint moments. We hypothesize that older adults would have a stronger relationship between the MOS and hip joint moments than young adults during the single-stance phase. Further, we hypothesize that older adults would have a reduced MOS-AP and MOS-ML than young adults when assessed continuously over the entire stance phase.

## Methods

### Sample characteristics

Fourteen healthy older adults (66.9 ± 4.3yrs, 1.73 ± 0.08m, 76.9 ± 10.8kg) and sixteen healthy young adults (26.3 ± 3.6yrs, 1.72 ± 0.10m, 66.2 ± 14.0kg) were recruited from the greater St. Pölten (AUT) metropolitan area. Inclusion criteria included individuals aged between 60–75 years old for the older adults and 20–35 years old for the younger adults. Further, all participants were required to be able to walk independently without the use of external support. Exclusion criteria included any medically diagnosed diseases that affected gait, surgeries affecting the musculoskeletal systems in the last six months, musculoskeletal injuries in the past six months that affected gait, and medically diagnosed neurocognitive impairments. This study was approved by the Ethics Committee of Lower Austria in accordance with Declaration of Helsinki and all participants signed an informed consent prior to participating.

### Data collection and analysis

Participants walked along a 12m walkway at their preferred speed until three trials each containing one full gait cycle were collected by the force plates. Three-dimensional motion capture was conducted with a 16 camera Vicon system (Nexus, 2.14, Vicon, Oxford UK) and three force plates (AMTI, Watertown, MA) at 120Hz and 1200Hz, respectively. Participants were outfitted with a full-body marker set using the Cleveland Clinic marker set for the lower extremity and the Vicon Plug-In Gait model for the upper body [13]. Raw trajectory and ground reaction force data were filtered with a 4^th^ order low-pass Butterworth filter with a 10Hz and 15Hz cut frequency, respectively [9]. Data were then exported to Visual 3D (C-Motion, Germantown MD) for reconstruction as a 15-segment full-body model for 3D kinematic and kinetic calculations [23, 24]. The BOS per step was defined as the instantaneous COP position which provides a more accurate representation of the effective BOS [15, 25]. The COM, COM velocity, COP, and joint moments (normalized to body mass) were calculated in Visual 3D prior to processing in custom Matlab 2023b (Mathworks, Natick MA) scripts for further analyses. The MOS was calculated in the sagittal and frontal planes for each foot with the formula:

MOS=COP–xCOM

Where xCOM is the extrapolated COM calculated as:

xCOM=COMp+(COMv/ωθ)

In which COMp = COM position and COMv = COM velocity. ωθ was calculated as:

ωθ=√g/l

In this term, g = 9.81m/s^2^ and l is the length of the inverted pendulum determined as the average distance of the right/left lateral heel marker to the COM [15]. Data for the left and right legs were averaged across the three trials per participant. MOS and joint moments were calculated in the sagittal and frontal planes. For the knee joint, only sagittal plane joint moments were assessed. All data were time normalized to 101 points. Joint moments were calculated across the gait cycle and per step while the MOS was calculated only per step.

## Statistical analyses

Data were statistically analyzed in Matlab 2023b (Mathworks, Natick, MA). The normality of variables was verified using a Shapiro-Wilk’s test prior to running independent t-tests using the SPM1D package (v.0.4.2) with statistical significance at p < 0.05 [26, 27] on the MOS and joint moment waveforms in both the sagittal and frontal planes. To assess the relationship between the MOS and joint moment waveforms in each plane, the cross-correlation was assessed with peaks extracted at a zero-phase lag during the single-stance phase [28]. The cross-correlation quantifies the correlation between two continuous signals by shifting one signal along a time delay continuum against the stationary second signal [28]. This process returns correlation values along each time delay which ranges from +1 to –1 with a phase-lag of zero representing no temporal shift. While previous research has either extracted absolute correlation peaks across all time shifts or from peaks near the time delay of zero, we extracted the peaks at the zero-phase lag due to our signals being already time normalized [28]. For the cross-correlation analysis, we correlated the time normalized signals (101 points) of joint moments and MOS, both of which were extracted during the single-support phase.

Cross-correlation values were considered strong if they were greater than 0.5, moderate 0.3–0.5, and weak 0.0–0.3. Negative values of this range of values were used to showcase a potential negative relationship between the signals. Cross-correlations were calculated per participant in each group. An independent t-test was then used to assess between group differences in the strength of the cross-correlation for each MOS and joint moment pairing. To account for multiple comparisons in the cross-correlation values, a false discovery rate (FDR) correction (i.e. Benjamini-Hochberg) was used.

## Results

No group differences in walking speed, step time, step length, and step width were found (p > 0.05), **Table 1**. In the sagittal plane, a strong relationship for the hip-MOS occurred for the young (r = 0.87) and older (r = 0.91) adults with the t-test on the cross-correlation values revealing that the older adults had a stronger correlation (t(1,28) = 2.83, p < 0.009) than the younger. Further, a weak relationship for the knee-MOS for young (r = 0.38) and older (r = 0.37), and a strong relationship for the ankle-MOS for young (r = -0.82) and older (r = -0.85) adults, **Table 2**, occurred. After post-hoc comparisons, only the hip-MOS relationship remained significant between groups.

**Table 1:** Average and standard deviation (SD) for walking speed and spatiotemporal parameters for older and younger adults.

	Older Adults	Young Adults
Mean ± SD		
Walking Speed (m/s)	1.17±0.22	1.19±0.17
Step Time (s)	0.56±0.05	0.54±0.05
Step Length (m)	0.61±0.06	0.64±0.07
Step Width (m)	0.08±0.03	0.08±0.03

**Table 2:** Average and standard deviation (SD) for cross-correlation between joint moments and the Margin of Stability per plane for older and younger adults. False Discovery Rate P-values are reported.

	Older Adults R-Value	Young Adults R-Value	P-value
Mean ± SD			
Frontal Plane Hip-MOS	0.49±0.11	0.38±0.17	0.062
Frontal Plane Ankle-MOS	-0.22±0.38	0.09±0.38	0.062
Sagittal Plane Hip-MOS*	0.91±0.02	0.87±0.05	0.045
Sagittal Plane Knee-MOS	0.37±0.13	0.37±0.08	0.93
Sagittal Plane Ankle-MOS	-0.85±0.06	-0.82±0.04	0.14

* statistical significance between groups (p < 0.05)

**Fig 1:**
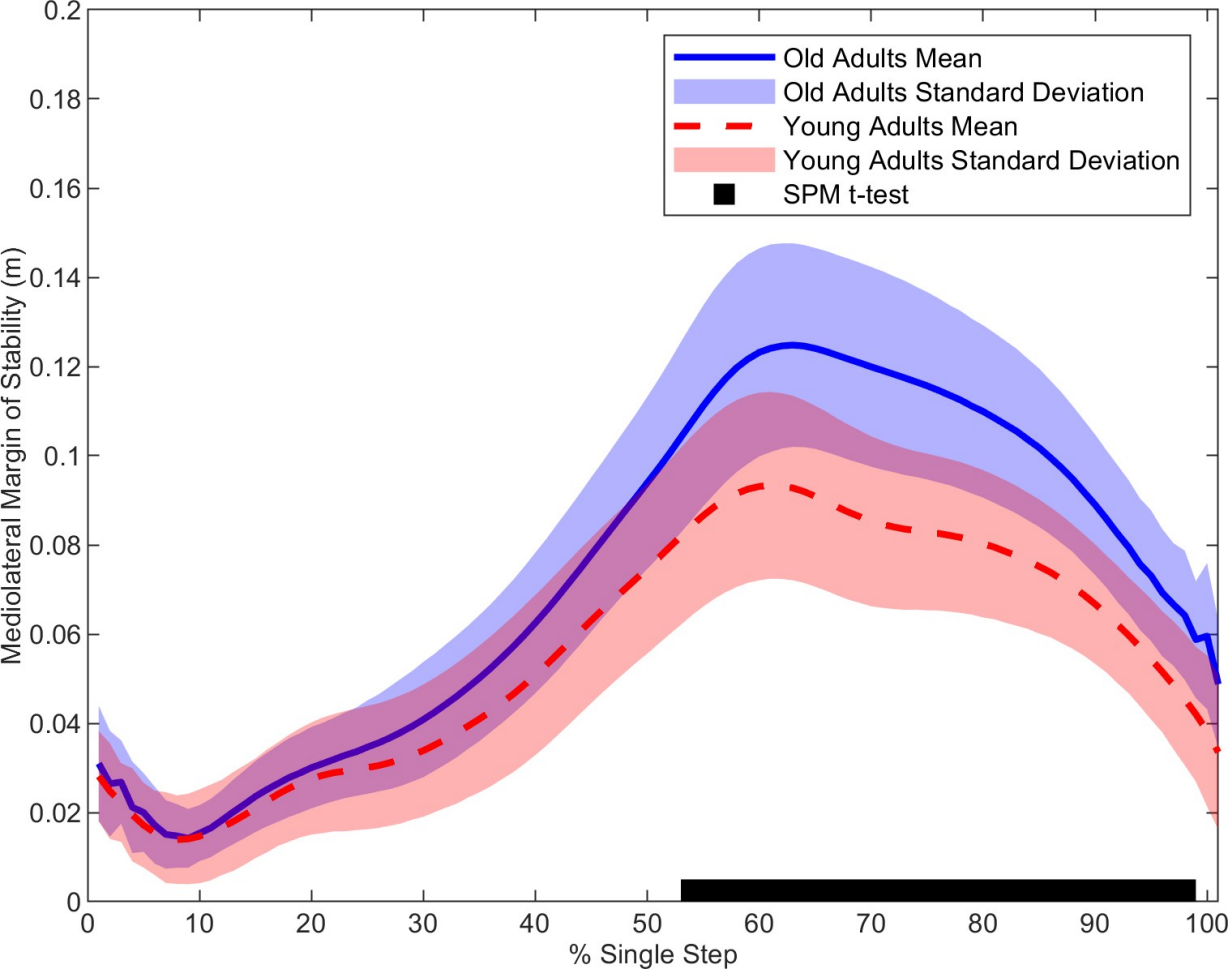
The average mediolateral Margin of Stability time-normalized from initial-contact to toe-off for healthy young (red) and older (blue) adults. Statistical differences between groups throughout the time normalized step are denoted along the x-axis with the black bar.

**Fig 2:**
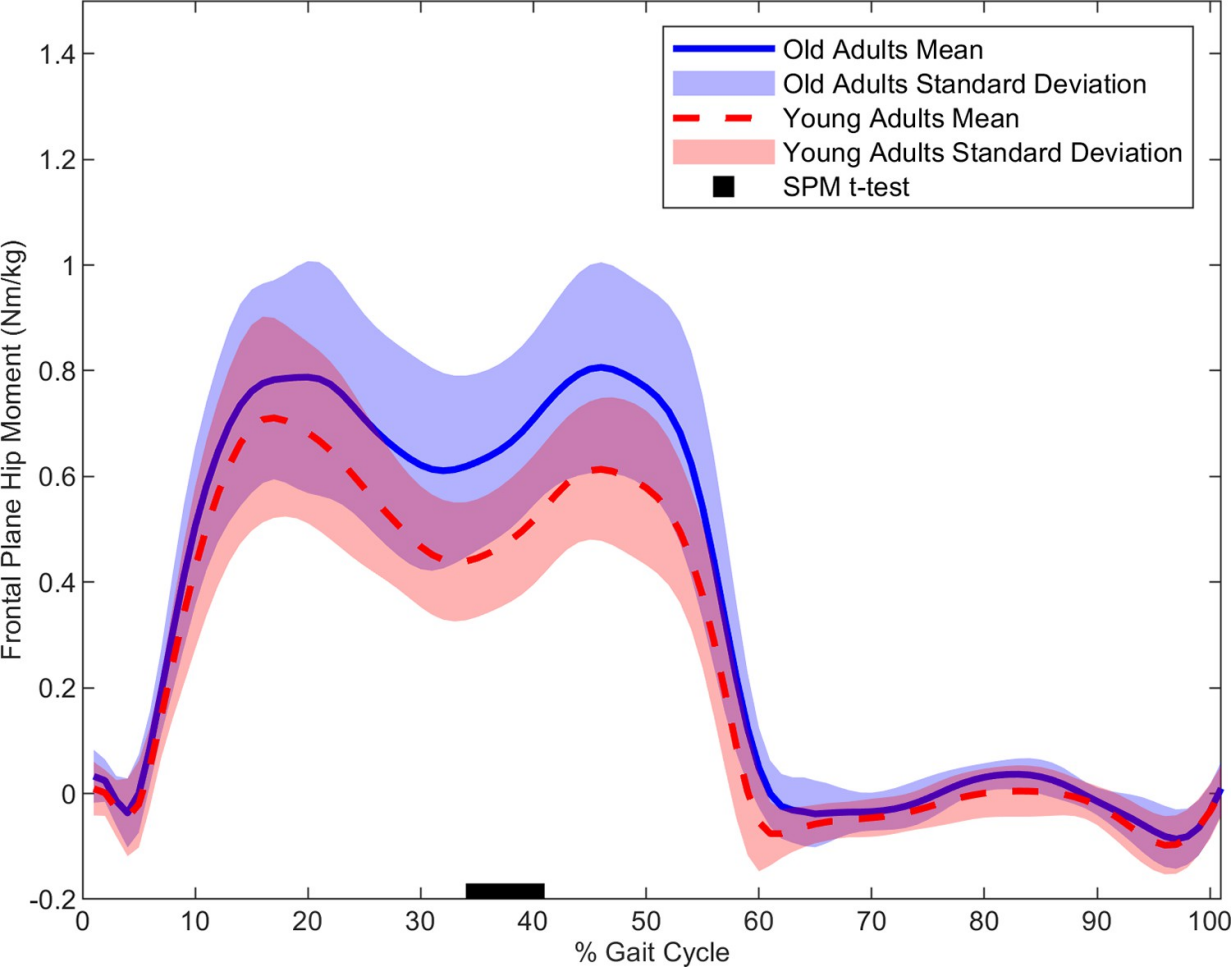
The average hip moment in the frontal plane time normalized to the gait cycle for young (red) and older (blue) adults. Statistical differences between groups throughout the time normalized step are denoted along the x-axis with the black bar.

**Fig 3:**
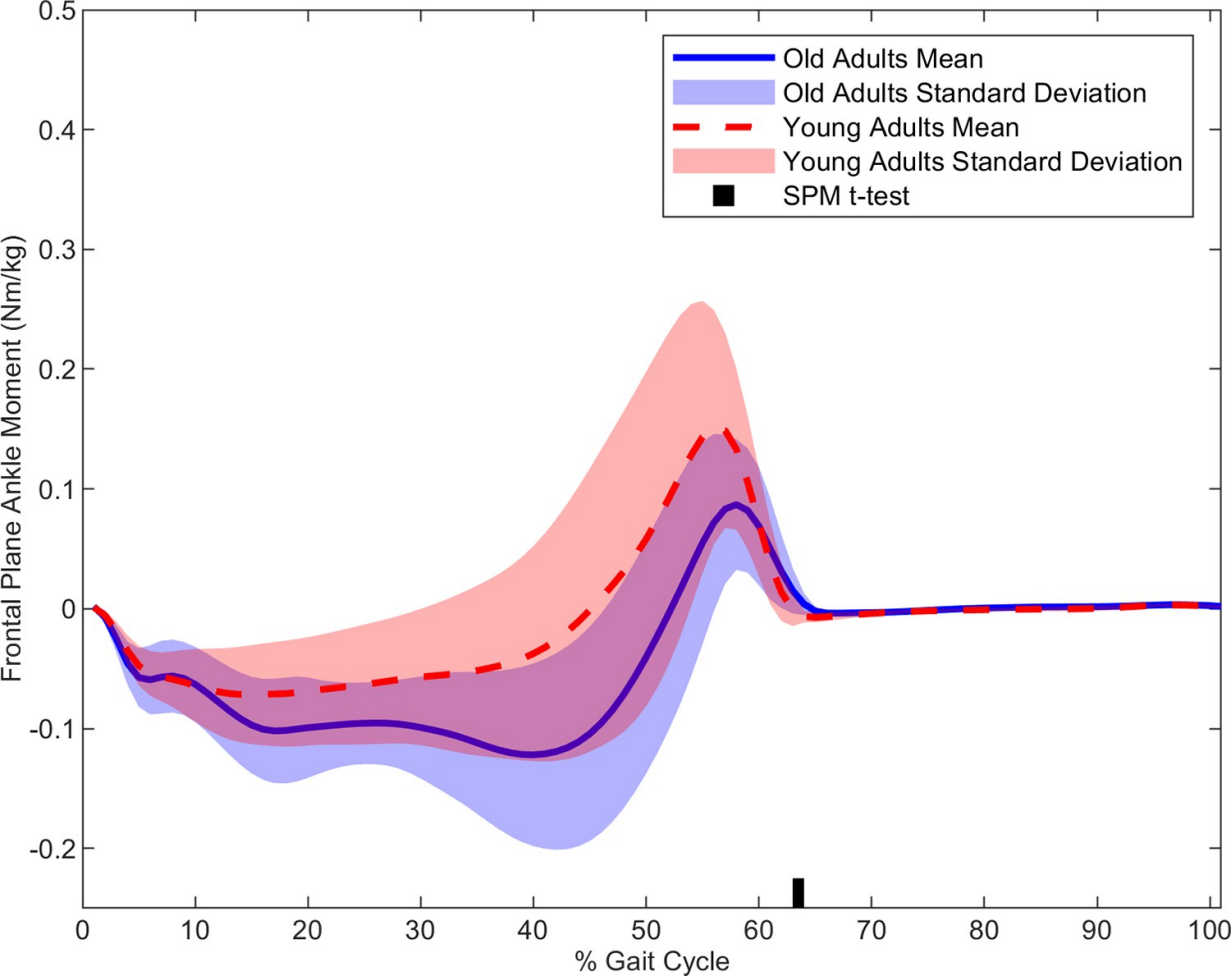
The average ankle moment in the frontal plane time normalized to the gait cycle for young (red) and older (blue) adults. Statistical differences between groups throughout the time normalized step are denoted along the x-axis with the black bar.

In the frontal plane, older adults had a larger MOS (t(1,28) = 3.01, p < 0.001) (Fig 1), larger hip abduction moment (t(1,28) = 3.48, p = 0.001) (Fig 2) and larger ankle eversion moment (t(1,28) = 3.55, p < 0.037) (Fig 3) than the younger adults. Cross-correlation demonstrated a moderate relationship for the young (r = 0.37) and older adults (r = 0.49) for the hip-MOS pairing, **Table 2**, respectively. The t-test on the cross-correlation values revealed a stronger relationship for the older adults than the young (t(1,28) = 2.19, p < 0.037) for the frontal plane hip-MOS pairing. A very weak relationship occurred between the ankle-MOS for young (r = 0.09) and older (r = -0.22) adults, **Table 2**. The t-test revealed a stronger relationship for the older adults than the young (t(1,28) = -2.20, p < 0.036) for the frontal plane ankle-MOS pairing. However, statistical significance was not maintained for cross-correlation values after the FDR adjustments. No further statistical significances were found. All non-significant time normalized curves for the remaining outcome parameters can be found in the *Supplementary Material*.

## Discussion

### Main findings

This study examined if healthy aging affects the relationship between the MOS and leg joint moments in healthy young and older adults. Contrary to our hypothesis, older adults had a larger MOS in the frontal plane than the younger adults while demonstrating no differences in the sagittal plane for this metric. However, in line with our hypothesis, older adults had a stronger relationship between the MOS and hip joint moments in the sagittal plane than the younger adults. The stronger relationship in the sagittal plane between the MOS and the hip joint moment for older adults may indicate that healthy aging increases the coupling between the hip moments and the COM to control dynamic stability during single-support. Additionally, the larger frontal plane hip abduction moment observed in our older adults, compared to young adults may reflect an effort to control the trajectory of the xCOM in the frontal plane as it moves away from the BOS.

### Margin of Stability

In contrast to our hypothesis, the healthy older adults in our study had a larger MOS in the frontal plane than the younger adults during the single-support and the beginning of the second double-support phases. This finding is unexpected as a larger value for the MOS in the frontal plane is suggested to indicate greater instantaneous dynamic stability [15, 18, 29–31]. Specifically, previous literature proposes that the further medial the xCOM is from the lateral edges of the BOS (indicated by a larger and more positive MOS), the greater the level of dynamic stability is in the frontal plane [15, 30, 32]. Key methodological differences may account for this discrepancy.

For instance, research thus far quantified the MOS in the frontal plane at discrete time points in the gait cycle (initial-contact and toe-off) and/or utilized foot markers to define the BOS lateral boundaries [16, 17, 21, 22, 30, 31, 33, 34]. Several limitations exist in the quantification and interpretation of the MOS when exclusively examining initial-contact and toe-off. Specifically, initial-contact occurs during the double-support phase when the BOS is largest and corrective action to adjust potential errors in the trajectory of the COM can be larger [6, 9, 10]. Indeed, as both feet are on the ground, the location of the COP can be shifted over a longer and wider surface area to adjust the location of the COM after foot placement is achieved [6, 9, 10]. This corrective action, however, is more limited during the single-support phase as the BOS of the single foot is shorter and narrower [6, 9, 10]. This poses a particular challenge to the neuromuscular system when maintaining dynamic stability as the COM reaches its most lateral position during the single-support phase [9, 10]. Therefore, to control the large inertial load of the upper body, the neuromuscular system generates stabilizing moments from the ankle and hip to avoid a loss of balance [10]. However, the effect of these moments on the MOS would require time for execution and would not necessarily be quantifiable at the exact instant of toe-off. Additionally, previous research has predominately utilized foot markers to define the BOS which may underrepresent the COP excursion underneath the foot during the gait cycle which, in turn, directly impacts the quantification of the MOS [25]. Therefore, in both cases, critical information relating to how aging affects the mechanisms contributing to dynamic stability and fall risk in older adults may be overlooked. Only by quantifying the MOS across a step with the excursion of the COP did we find differences between the older and younger adults.

The larger frontal plane MOS in our older adults compared to the younger adults, however, raises the notion that the MOS quantifies a neuromuscular strategy aimed at avoiding loss of dynamic stability rather than a quantification of an individual’s mechanical stability level that would reflect fall risk. Specifically, the larger frontal plane MOS in our older adults may indicate attempts to reduce the likelihood of lateral balance loss by shifting their xCOM away from the lateral boundaries of their BOS. While this strategy may be aimed at mitigating lateral balance loss, the xCOM is brought closer to the medial borders of the BOS which may cause pre-mature termination of the contralateral swing phase if medially directed destabilization occurs. Therefore, future research on falls should consider examining the effect of mediolateral perturbations applied during the single-support phase to determine whether this motor strategy leads to a heightened fall risk in older adults.

Unlike the frontal plane, no group differences were observed in the sagittal plane for the MOS. The lack of differences in this plane may be because of the distinct neuromuscular mechanisms that control dynamic stability in the sagittal compared to frontal plane [5]. Control of the COM in the sagittal plane while walking is considered predominately regulated by automated sub-cortical and somatosensory mechanisms [5, 35]. In contrast, the mechanics of the frontal plane are controlled by active mechanisms that stem from higher level cortical regions [5, 35]. Within this multifaceted control system, current evidence suggests that the active mechanisms which control frontal plane mechanics are particularly sensitive to the neurodegeneration that occurs from healthy aging while the sub-cortical mechanisms remain relatively unaffected [5]. Indeed, Bruijn et al. [36] found that a lower white matter microstructural organization in cortices of older adults, compared to younger adults, was strongly correlated with narrower steps and a smaller MOS in the frontal plane during treadmill walking. Thus, in our study, no sagittal plane differences in the MOS further supports the notion that healthy aging has a more limited impact on dynamic stability in the plane of progression [5, 36]. Instead, the aging neuromuscular system may strive to execute a more conservative control of the COM in the frontal plane during overground walking by maintaining their upper body closer to the center line of progression. This may be a preemptive strategy to potentially curb the neurodegenerative effects on the active mechanisms that control frontal plane dynamic stability during overground walking.

As such, future research should consider examining the allocation of attentional resources, which stem from higher level cortical regions (ex: pre-frontal, frontal, and primary motor cortices), over the full gait cycle to reveal underlying compensation strategies implemented by healthy older adults. While the literature on dual-tasking is extensive, the evidence thus far is sparse in examining the effects of a secondary dual-task on dynamic stability when assessed with statistical parameter mapping [37, 38]. Additionally, further research should consider applying neuroimaging to these dual-tasking protocols to provide direct evidence for changes related to the allocation of attentional resources when controlling dynamic stability in healthy older adults.

### Joint kinetics

Our hypothesis was partially supported as the older adults had a stronger correlation, than the younger adults, between the hip joint moment and the MOS only in the sagittal plane. This finding may indicate that, in the sagittal plane, older adults exert a tighter control on their xCOM to attenuate the destabilizing forces acting on the upper body. Although no group differences were observed in the relationship between the MOS and the joint moments in the frontal plane, the older adults had a larger frontal plane hip moment during the single-stance phase than the younger adults.

In the sagittal plane, the hip joint reaction force is the primary destabilizing force which causes the upper body to flex during weight-acceptance and extend during push-off [6]. Further, during the swing phase, the xCOM is held in front of the BOS to progress the body forward [5, 6, 9, 13]. As the BOS is located behind the xCOM, corrective action by the BOS to contribute to one’s dynamic stability level is only feasible by effective foot placement at upcoming initial contact [6, 8, 9]. Therefore, to control the upper body’s movement, the musculature of the hip executes an opposing moment to maintain upright posture and dynamic stability during the single-support phase [6]. In our study, the role of the hip muscles in controlling dynamic stability would account for the very strong relationship between these parameters in the sagittal plane for both groups. Furthermore, the stronger relationship for these parameters in our older, than younger, adults is in line with the relatively greater contribution from their hip than ankle moments in this demographic while walking [14]. However, the group difference in the sagittal plane hip and MOS pairing was relatively small and did not coincide with any additional group differences in this plane. This may indicate that older adults, compared to young, increase the coupling between the hip and upper body to maintain dynamic stability, without the need to increase the generation of joint moments at the hip.

In contrast to the sagittal plane, gravity is the primary destabilizing force in the frontal plane causing the upper body to accelerate medially toward the center line of progression during the single-support phase [10]. Thus, to avert a fall, the hip abductors generate a nearly equal and opposite counterbalancing moment to control the medial acceleration of the COM [6, 10]. Previous research suggests that this careful balancing of moments depends on the ability of the somatosensory system to sense the kinematic state of the COM during the stance-phase, so that the neuromuscular system can then generate an effective counterbalancing hip moment [6, 10]. This intricate system then maintains an individual’s erect posture and reduces the likelihood of a mediolateral balance loss or the need for corrective stepping [6, 9, 10]. In relation to our study, the increased hip moment and frontal plane MOS for older than younger adults occurred during the single-stance phase. This may indicate that, compared to the young adults, our older adults increased their hip abduction moment to better control the larger medial acceleration of the xCOM while their MOS in the frontal plane is enlarged.

The increased hip abduction moment was, however, brief despite the continued enlargement of the frontal plane MOS into the double-support phase in our older adults. One possible explanation may be that the purpose of the brief increase in the hip abduction moment was to mitigate small deviations from the nominal trajectory of the xCOM in the frontal plane as it separated from the BOS of the stance limb. Indeed, in the frontal plane, small deviations to the nominal trajectory of the upper body exist and must be constantly attenuated by the neuromuscular system [5, 29]. Previous research demonstrates that such deviations also arise during unperturbed walking due to the natural variability in an individual’s gait pattern [5, 29, 39]. In older adults, compared to young, current evidence demonstrates a further increase in the deviations to this nominal trajectory which has been linked to age related increases in gait variability [5, 40–42]. Therefore, in our study, the brief increase in the hip abductor moment in our older adults may have served to attenuate potentially deviations in the monophasic trajectory of the xCOM during the gait cycle [5, 6, 10].

It should be noted, however, that a certain amount of natural variability is still necessary in this control system to facilitate gait adaptation to environmental demands [5, 39]. This would account for the moderate relationship between the hip and MOS in the frontal plane for both groups in our study. The moderate relationship between these parameters may indicate a control system whereby the medial acceleration of the COM is flexibly regulated to maintain its trajectory toward to its position at upcoming initial contact. Further, as the kinematic state of the COM is the primary determinant of step width, a stringently tight system would reduce the natural variability in the neuromuscular system which facilitates gait adaptation to changes in environmental demands [5, 31, 39, 43].

### Limitations

When considering the findings of our research several limitations should be considered. In our study, we did not record participants’ fall history nor fear of falling. Both aspects are shown to cause changes in the gait pattern in older adults. Thus, we are unable to rule out any potential adjustments or impacts fall history or fear of falling have on our findings. Additionally, by examining differences between healthy younger and older adults, our results do not examine at which point during the adult lifespan that changes to control of the frontal plane MOS begin to occur. Finally, we conducted our analysis on a convenience sample which may have limited our ability to detect all potentially significant differences between older and younger adults.

## Conclusion

Our findings indicate that healthy aging increases the MOS in the frontal plane during the single-support phase and at the beginning of the double support phase. This finding has strong implications for fall research and fall prevention therapies for older adults, as a larger MOS has been previously interpreted as indicative of greater dynamic stability. Thus, a paradox arises with this definition whereby older adults who have a heightened fall risk would have a larger dynamic stability quantification—indicative of a reduced fall risk—than younger adults. In contrast to this definition, our results may indicate that in healthy older adults the MOS quantifies a motor strategy that attempts to reduce the likelihood of a lateral balance loss rather than reflecting an individual’s mechanical fall risk level. Specifically, this motor strategy may reflect attempts to compensate for the neurodegeneration of the active neuromuscular mechanisms that control frontal plane dynamic stability. Our results on the MOS additionally highlight the importance of quantifying the MOS over the entire single-stance phase, rather than at discrete time points in the gait cycle, when examining fall risk. In doing so, underlying differences related to dynamic stability in fall-prone populations may be revealed which otherwise could be masked by the lack of continuous data analyses. Finally, our results also indicate that healthy aging increases the coupling between the MOS and the hip moment in the sagittal plane during the single-support phase. This plausibly was an active effort by the neuromuscular system to control upper body movement to attenuate the destabilizing effects from the hip joint reaction forces in the plane of progression. The increased coupling between the sagittal plane hip moment and MOS plausibly stemmed from the distal-to-proximal redistribution of joint moments which occurs during healthy aging.

## Supporting information

S1 Fig The average sagittal plane Margin of Stability time-normalized from initial-contact to toe-off for healthy young (red) and older (blue) adults. (TIF)

S2 Fig The average hip moment in the sagittal plane time normalized to the gait cycle for young (red) and older (blue) adults. (TIF)

S3 Fig The average knee moment in the sagittal plane time normalized to the gait cycle for young (red) and older (blue) adults. (TIF)

S4 Fig The average ankle moment in the sagittal plane time normalized to the gait cycle for young (red) and older (blue) adults. (TIF)
