# Supplementary figures and images for: Mediolateral Margin of Stability highlights motor strategies for maintaining dynamic balance in older adults
Source: PLoS One. 2024 Oct 31;19(10):e0313034. doi: 10.1371/journal.pone.0313034 (PMC11527164; doi:10.1371/journal.pone.0313034)

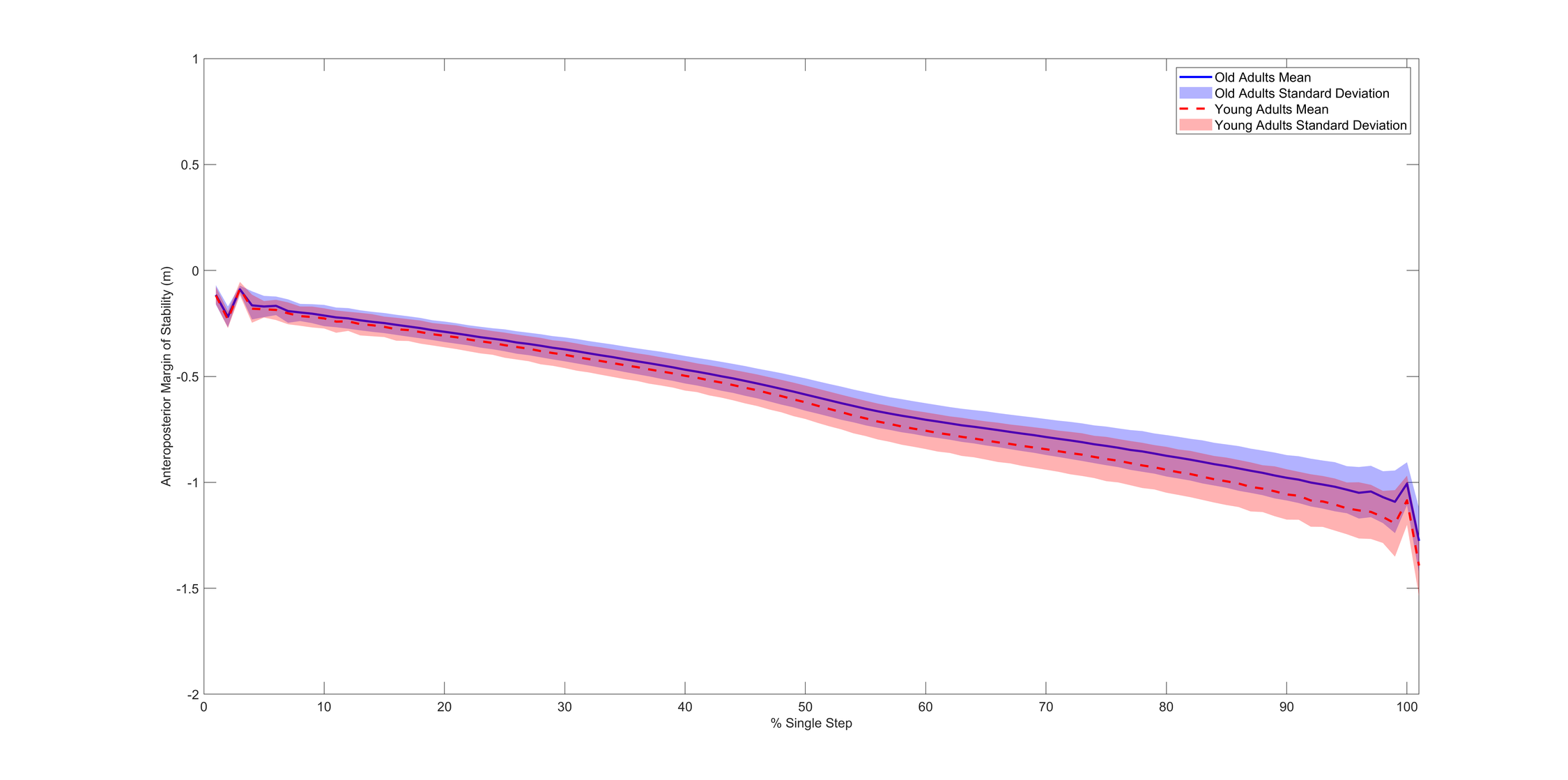

Supplement: S1 Fig — (TIF) [file pone.0313034.s001.tif]

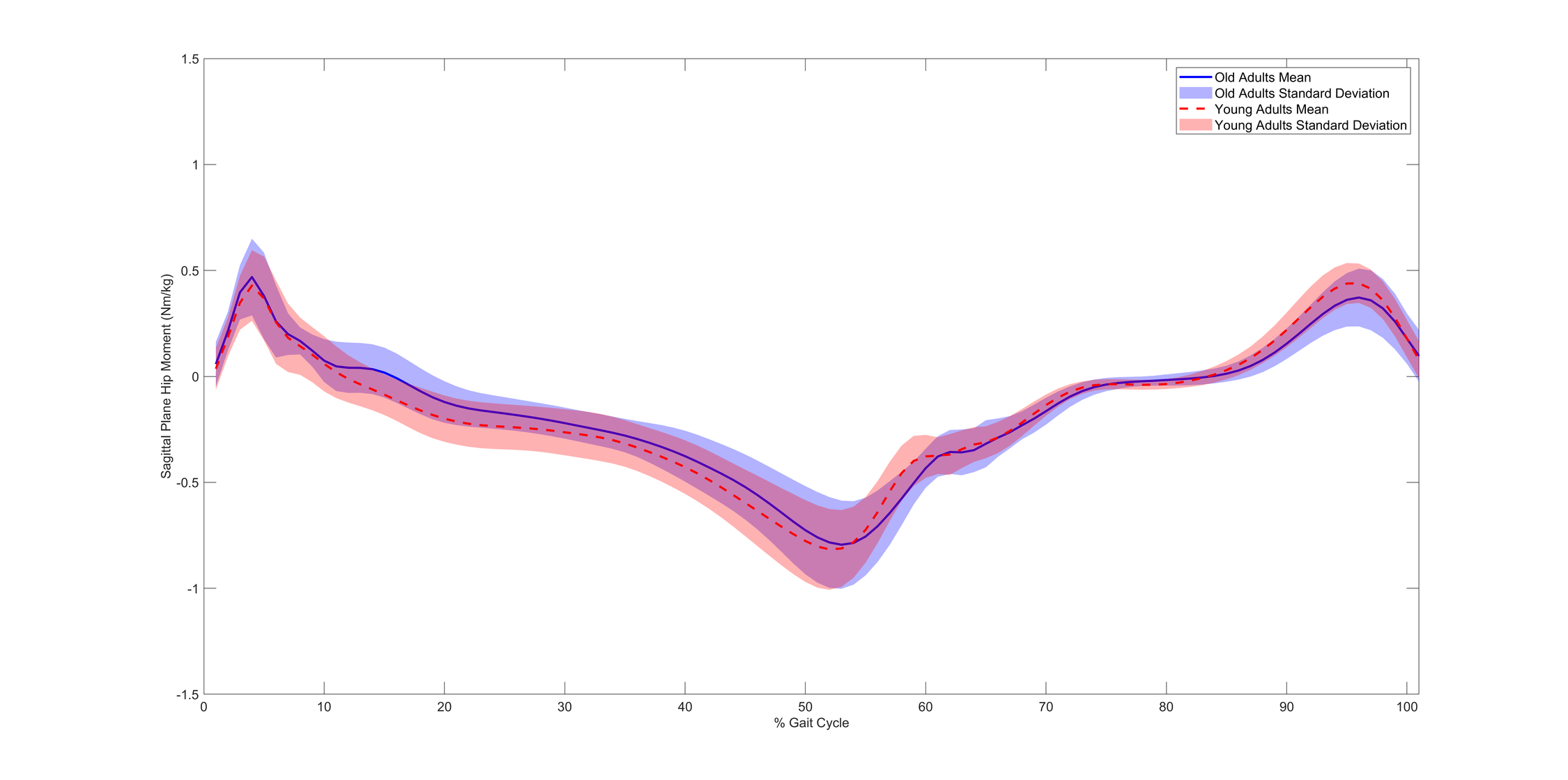

Supplement: S2 Fig — (TIF) [file pone.0313034.s002.tif]

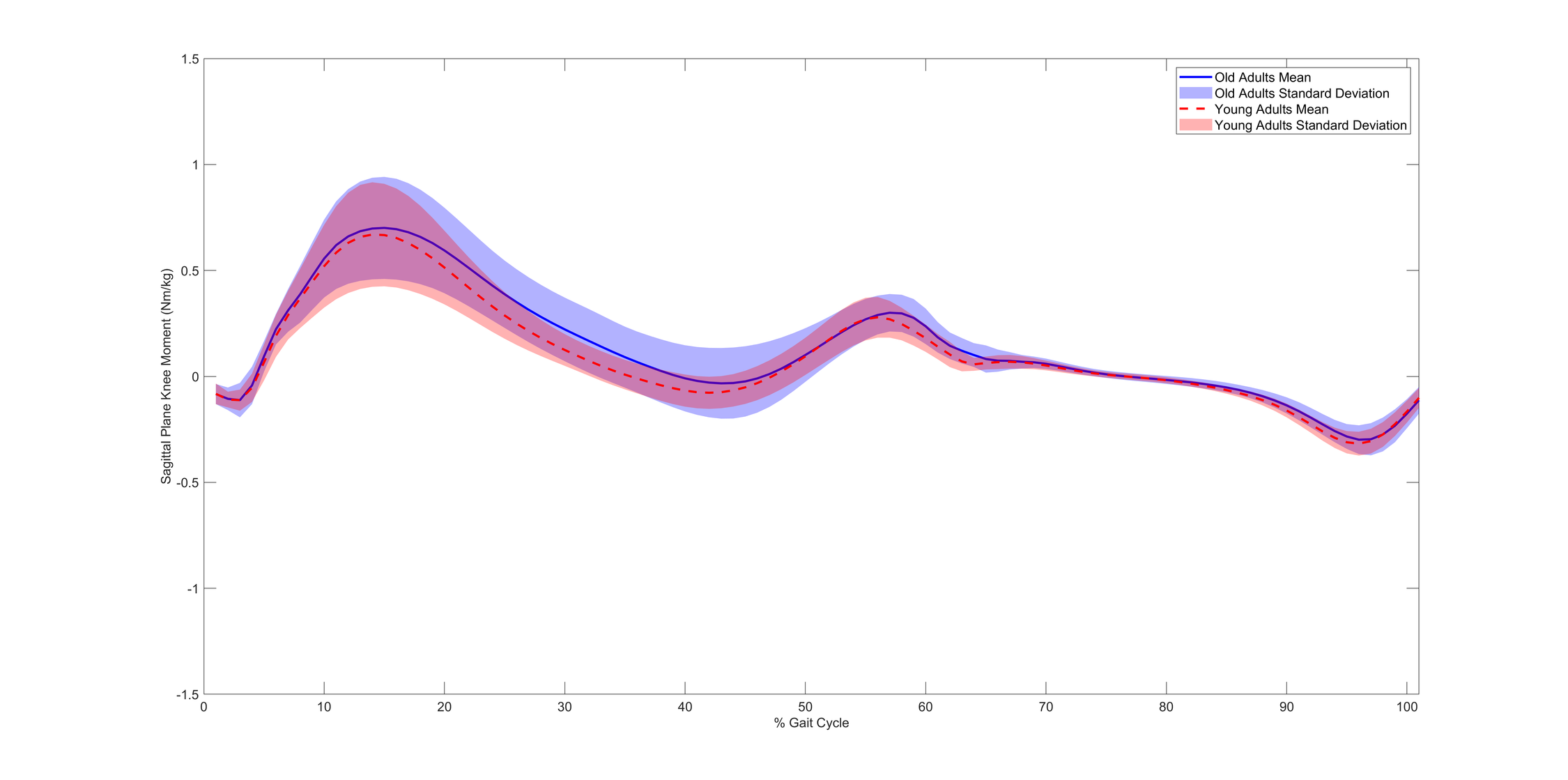

Supplement: S3 Fig — (TIF) [file pone.0313034.s003.tif]

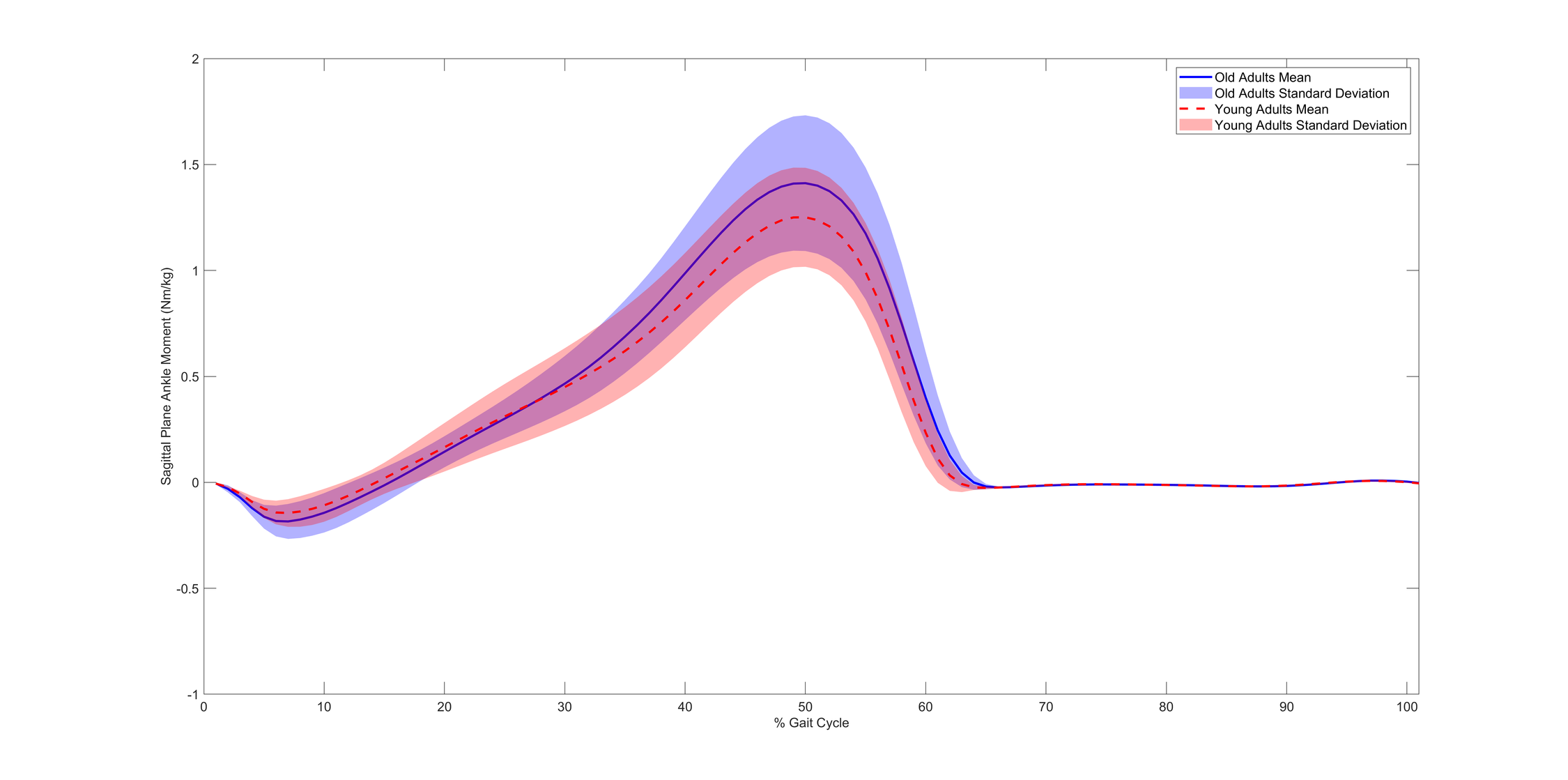

Supplement: S4 Fig — (TIF) [file pone.0313034.s004.tif]
